# Phenotypic and Spatial Characterization of Tumor-Associated Macrophages in Non-Metastatic Seminoma: Association with Local Tumor Progression

**DOI:** 10.3390/medsci13030129

**Published:** 2025-08-14

**Authors:** Grigory Demyashkin, Vladimir Shchekin, Dmitriy Belokopytov, Tatyana Borovaya, Ivan Zaborsky, Kadir Safiullin, Oleg Karyakin, Alexey Krasheninnikov, Nikolay Vorobyev, Petr Shegay, Andrei Kaprin

**Affiliations:** 1 Department of Digital Oncomorphology, National Medical Research Centre of Radiology, 2nd Botkinsky Pass, 3, 125284 Moscow, Russia; dr.shchekin@mail.ru (V.S.); beldimbur@gmail.com (D.B.); tbor27@yandex.ru (T.B.); i.zaborskii@mail.ru (I.Z.); skadir@mail.ru (K.S.); karyakin@mrrc.obninsk.ru (O.K.); krush07@yandex.ru (A.K.); dr.vorobyev@mail.ru (N.V.); dr.shegai@mail.ru (P.S.); kaprin@mail.ru (A.K.); 2 Laboratory of Histology and Immunohistochemistry, Institute of Translational Medicine and Biotechnology, I.M. Sechenov First Moscow State Medical University (Sechenov University), Trubetskaya Str. 8/2, 119048 Moscow, Russia; 3 Research and Educational Resource Center for Immunophenotyping, Digital Spatial Profiling and Ultrastructural Analysis Innovative Technologies, Peoples’ Friendship University of Russia (RUDN University), Miklukho-Maklaya Str.6, 117198 Moscow, Russia; 4 Department of Urology and Operative Nephrology, Peoples’ Friendship University of Russia (RUDN University), Miklukho-Maklaya Str.6, 117198 Moscow, Russia

**Keywords:** seminoma, macrophages, TAMs, M1 and M2 phenotypes, CD68 and CD163

## Abstract

**Background:** Seminoma is the most common subtype of testicular germ cell tumors in young men; however, the contribution of tumor-associated macrophages (TAMs) to disease progression remains insufficiently understood. This study aimed to quantitatively and phenotypically characterize CD68^+^ and CD163^+^ TAMs in non-metastatic seminomas (pT1N0M0 and pT2N0M0). **Methods**: This retrospective, multicenter, cohort, observational, analytical study was conducted from 1 January 2015 to 1 January 2025 at two branches of the National Medical Research Radiological Center of the Ministry of Health of the Russian Federation: the A. Tsyb Medical Radiological Research Center and the P. Hertsen Moscow Oncology Research Institute. Archived paraffin-embedded tumor samples from 96 patients and 21 samples of normal testicular tissue were analyzed using immunohistochemistry and digital morphometric analysis with QuPath software to assess macrophage density and spatial distribution. **Results**: Compared to normal testicular tissue, seminomas demonstrated more than a 10-fold increase in CD68^+^ TAMs and over a 100-fold increase in CD163^+^ TAMs. CD68^+^ cells predominantly localized to peripheral tumor regions, while CD163^+^ cells formed diffuse clusters in central tumor zones and around peripheral vessels. No statistically significant differences in CD68^+^ cell density were found between pT1 and pT2 stages. However, pT2 tumors showed a trend toward higher CD163^+^ TAMs density, suggesting increased M2 polarization with advancing tumor stage. **Conclusions**: These findings highlight the spatial and phenotypic heterogeneity of TAMs in seminoma and indicate a shift toward an immunosuppressive tumor microenvironment during local progression. Future studies should assess macrophage polarization and progression-free survival to evaluate their potential as prognostic biomarkers and therapeutic targets in seminoma.

## 1. Introduction

Germ cell tumors (GCTs) represent the most common malignancies in young men, predominantly between the ages of 15 and 40, despite accounting for a relatively small proportion of all cancers. Globally, approximately 74,000 new cases of GCTs are diagnosed annually, with the highest incidence rates observed in Northern and Western European countries, reaching up to 9–12 cases per 100,000 men. Seminoma is the most prevalent histological subtype of GCTs, comprising approximately 55–60% of cases, with a peak incidence between 35 and 45 years of age. Most seminomas (up to 80%) are diagnosed at early stages (pT1N0M0/pT2N0M0) [1].

Seminoma is associated with excellent overall survival rates, reaching up to 99%. Disease classification follows the pTNM system, ranging from stage IA to IIIC, with additional stratification based on tumor marker levels—namely of human chorionic gonadotropin (hCG), alpha-fetoprotein (AFP), and lactate dehydrogenase (LDH) [2]. According to current clinical guidelines, standard treatment includes radical inguinal orchiectomy, with adjuvant chemotherapy and/or radiotherapy administered when indicated, particularly in cases with regional lymph node metastasis [3].

Despite significant advances in the molecular and genetic understanding of seminomas, the interaction between tumor tissue and the tumor microenvironment (TME) remains poorly understood. A distinctive feature of seminomas compared to other GCTs is the abundant infiltration of immune cells, suggesting an important role for the TME in disease progression [4,5,6].

The testis is known for its immune-privileged status, maintained by the blood–testis barrier, which prevents autoimmune reactions against spermatogenic cells. However, inflammatory and neoplastic processes can disrupt this tolerance, leading to the infiltration of immune-competent cells, including macrophages, dendritic cells, and T lymphocytes. Some studies have reported that non-metastatic seminomas display a more pronounced immune cell infiltration; for instance, the number of activated T cells (FOXP3^+^, PD-1^+^) is significantly higher compared to metastatic counterparts [7,8,9].

Histopathological remodeling of the immune landscape in seminomas involves complex interactions between mast cells, T and B lymphocytes, macrophages, atypical spermatogenic cells, and other somatic cells. These interactions may contribute to the unique immunophenotypic profile of seminomas and are thought to play a role in disease progression, including interactions with PD-1 and PD-L1 receptors [10,11].

Tumor-associated macrophages (TAMs) play a central role in the development of the tumor stroma and can constitute up to 50% of the cells within this stromal compartment. These cells exhibit marked functional plasticity and are capable of polarizing into different phenotypes—primarily the classically activated M1 phenotype and the alternatively activated M2 phenotype [12].

M1 macrophages possess antitumor properties: they produce pro-inflammatory cytokines (IL-1β, TNF-α, IL-6), enhance the Th1 immune response, and promote cytotoxic activity against tumor cells. In contrast, M2 macrophages are associated with immunosuppression and tumor progression; they are involved in tissue remodeling, angiogenesis, and the stimulation of tumor growth [13].

Cytokines secreted by tumor cells and Th2 lymphocytes (such as IL-4, IL-10, and TGF-β) drive the polarization of TAMs toward the M2 phenotype, resulting in immune suppression and accelerated tumor development. A high infiltration of M2-polarized TAMs has been linked to poor prognosis and increased metastatic potential in various solid tumors [14].

In the present study, we hypothesized that a quantitative increase in CD163^+^ tumor-associated macrophages (TAMs) in pT2-stage seminoma compared to pT1 may represent a clinically relevant factor, reflecting a shift toward an immunosuppressive tumor microenvironment at a more advanced stage of disease progression.

The clinical relevance of this hypothesis lies in the current management of patients with stage I seminoma following orchifuniculectomy. These patients are stratified into relapse risk groups, and decisions regarding adjuvant chemotherapy are based on the assigned risk category. Notably, patients in intermediate- and high-risk groups represent clinically ambiguous categories in which chemotherapy is not mandatory and remains a matter of clinical discretion. This underscores the need for additional stratification criteria. The evaluation of CD163^+^ TAM levels may complement existing risk factors and support more precise and personalized selection of adjuvant therapy [15].

Various approaches exist for determining TAM phenotypes, including immunohistochemical marker panels and molecular techniques such as quantitative real-time PCR (qRT-PCR). A wide range of markers has been described in the literature to distinguish M1- and M2-polarized macrophages. Markers commonly associated with the M1 phenotype include NOS2 (iNOS), TNF, IL1B, IL6, IL12A/B, CXCL9, CXCL10, IRF5, and STAT1, while M2 macrophages are characterized by the expression of ARG1, MRC1 (CD206), MSR1 (CD204), IL10, TGFB1, and STAT6. However, from both practical and economic perspectives, the implementation of extended marker panels in routine clinical practice remains challenging, and data interpretation may be difficult [16].

In this study, CD163 was selected as the most specific immunohistochemical marker for identifying M2-polarized TAMs. This marker demonstrated high sensitivity and reproducibility across multiple studies, and its expression is strongly associated with an immunoregulatory macrophage phenotype. Comparative analysis of the quantity and spatial distribution of CD163^+^ and CD68^+^ TAMs might have reflected a shift toward M2 polarization and have prognostic potential, making this approach not only pathophysiologically relevant but also clinically significant.

Thus, detailed investigation of the immunomorphological features of TAMs in non-metastatic seminoma represented a promising strategy for identifying novel prognostic markers, refining personalized therapeutic approaches, and enhancing our understanding of the role of macrophages in carcinogenesis.

According to the existing literature, the study by Sadigh et al. (2020) addressed PD-L1 expression and the tumor microenvironment, including CD68^+^ and CD163^+^ TAMs in germ cell tumors. In contrast, our study includes a substantially larger cohort of patients with non-metastatic seminoma and, for the first time, provides a comprehensive quantitative and phenotypic analysis of TAMs (CD68^+^/CD163^+^), including their spatial distribution across pT1 and pT2 stages. This approach reveals distinct patterns of macrophage infiltration and supports the potential use of M2 macrophages as a prognostic marker of local tumor progression. Further analyses are planned to explore TAM polarization in greater depth using multiplex immunohistochemistry and other advanced morphological techniques, as non-similar large-scale morpho-oncological investigations have previously been conducted in this context [17].

The aim of the present study was to compare the quantity and spatial distribution of TAMs (CD68^+^/CD163^+^) in non-metastatic seminoma at the pT1 and pT2 stages in order to assess their potential association with tumor progression.

## 2. Methods

### 2.1. Study Design and Setting

This retrospective, multicenter, cohort study was observational and analytical in design and was conducted from 1 January 2015 to 1 January 2025 at two branches of the National Medical Research Radiological Center of the Ministry of Health of the Russian Federation: the A. Tsyb Medical Radiological Research Center and the P. Hertsen Moscow Oncology Research Institute. Potential patient cases were initially identified through a search of the electronic medical record systems at both institutions using International Classification of Diseases, 10th Revision (ICD-10) codes corresponding to the primary diagnosis C62.1—Malignant neoplasm of descended testis.

The local ethics committee of the National Medical Research Center for Radiology approved this study (reference ID: 676) and waived the requirement for informed consent due to its retrospective and non-invasive nature. All samples and data were handled in strict accordance with the Guidelines on Human Biobanks and Genetic Research Databases, ensuring proper collection, anonymization, processing, and storage under internationally recognized ethical and quality standards. This article adhered to the Strengthening the Reporting of Observational Studies in Epidemiology (STROBE) guidelines. All procedures followed the principles of the Declaration of Helsinki.

### 2.2. Participant Selection

The study included patients (aged 20 to 63 years) who had undergone orchifuniculectomy with histologically confirmed seminoma classified as pT1N0M0 or pT2N0M0 and who had not received any prior systemic therapy (chemotherapy or radiotherapy). The diagnosis was confirmed morphologically and immunohistochemically using PLAP antibodies. Tumor staging was performed according to the pTNM classification (8th edition, UICC/AJCC). Based on clinicopathological data, patients were assigned to the following groups:

Group I (*n* = 96):

− Subgroup Ia: pT1N0M0 (*n* = 54);
− Subgroup Ib: pT2N0M0 (*n* = 42).

Group II (comparative group, *n* = 21): autopsy samples of morphologically normal testicular tissue obtained within 6 h after biological death. All individuals showed no macroscopic signs of tumor or inflammation. Causes of death included non-oncological and non-urogenital conditions (e.g., ulcerative bleeding, aneurysms, congenital anomalies, trauma). Each individual had fathered at least one biological child.

The research team reviewed electronic medical records to confirm eligibility criteria. Collected data included pTNM staging and clinicopathological characteristics. In cases of repeated hospital admissions, only primary tumor samples conforming to the study protocol were included in the analysis.

Patients were excluded from the study if they had evidence of metastatic disease (N+/M+), systemic conditions affecting immune tissue (e.g., sarcoidosis, systemic vasculitis), or unsuitable material (e.g., tissue degradation, lack of clinical data).

### 2.3. Outcome Measure

The primary endpoint was an increased density of CD163^+^ macrophages per mm^2^ in pT2-stage seminoma compared to pT1, as measured by immunohistochemistry and digital morphometry. The secondary endpoint was the spatial distribution of CD163^+^ TAMs.

### 2.4. Histologic Research

Serial 2 μm thick sections were obtained from archival paraffin-embedded tissue blocks of seminoma and normal testis. Sections were stained with hematoxylin and eosin (H&E). Histological analysis was performed using a Leica DM2000 light microscope with microphotography capabilities (Leica Microsystems, Wetzlar, Germany) in accordance with standard histopathological criteria.

### 2.5. Immunohistochemical (IHC) Assay

Immunohistochemistry was performed according to standard protocols. Primary antibodies used included anti-CD68 (Abcam; ab303565, dilution 1:1000, Cambridge, UK) and anti-CD163 (Abcam; ab182422, dilution 1:500, Cambridge, UK). For secondary antibody detection, the HiDef Detection™ HRP Polymer System (Cell Marque, Rocklin, CA, USA) was applied. This two-component system included anti-mouse/rabbit IgG antibodies, horseradish peroxidase (HRP), and DAB chromogen substrates. Cell nuclei were counterstained with Mayer’s hematoxylin.

Positive controls: Spleen tissue—rich in macrophages—was used for CD68, while tonsil tissue, known for strong macrophage staining, served as the positive control for CD163. Negative controls: Adjacent sections were stained without primary antibodies (using isotype-matched serum instead) to check for nonspecific background and chromogen reactivity. The staining patterns observed in control tissues closely matched those seen in the experimental seminoma samples, confirming the assay’s specificity and reliability.

### 2.6. Morphometric Study

Following digital slide scanning, the number of immunopositive cells was assessed via digital slide scanning using the Leica Aperio AT2 scanner. Quantification was performed using parametric analysis based on area (cells per mm^2^) and absolute cell counts. Image analysis was conducted using the open-source digital pathology software QuPath v0.5.1. To visualize the spatial distribution of CD68^+^ and CD163^+^ TAMs, density maps were generated using QuPath’s built-in functions with Gaussian kernel smoothing [18].

### 2.7. Data Collection

Data for this study were systematically extracted from the electronic medical records system. The collected data were then entered into a secure, encrypted Microsoft Excel spreadsheet (Microsoft Excel 2010; Microsoft Corporation, Seattle, WA, USA) for further processing and analysis. The spreadsheet was subsequently reviewed and verified by several co-investigators from the research team, who performed thorough checks to ensure the accuracy and completeness of the dataset before analysis. This multi-step verification process helped minimize errors and ensured the reliability of the data.

### 2.8. Statistical Analysis

Statistical analysis was performed using SPSS software, version 12.0 (IBM Analytics, New York, NY, USA). The Shapiro–Wilk test was used to assess the normality of data distribution. A *p*-value > 0.05 was considered indicative of a normal distribution. Since the data did not follow a normal distribution, intergroup comparisons of quantitative variables were conducted using the non-parametric Mann–Whitney U test. Data in tables are presented as median and interquartile range values (Me [Q1–Q3]). A *p*-value < 0.05 was considered statistically significant. Data visualization in the form of violin plots was carried out using the BioRender.com platform.

## 3. Results

### 3.1. Histological Patterns

All testicular samples from Groups I and II (*n* = 96; 100%) exhibited germ cell tumors (GCTs) with a lobular architecture and complete germ cell aplasia. The majority of atypical cells had abundant, clear cytoplasm with well-defined borders; their nuclei were polygonal and contained one or more prominent nucleoli. Tumor cells were arranged in nests or cords, separated by delicate fibrous septa.

**Figure 1:**
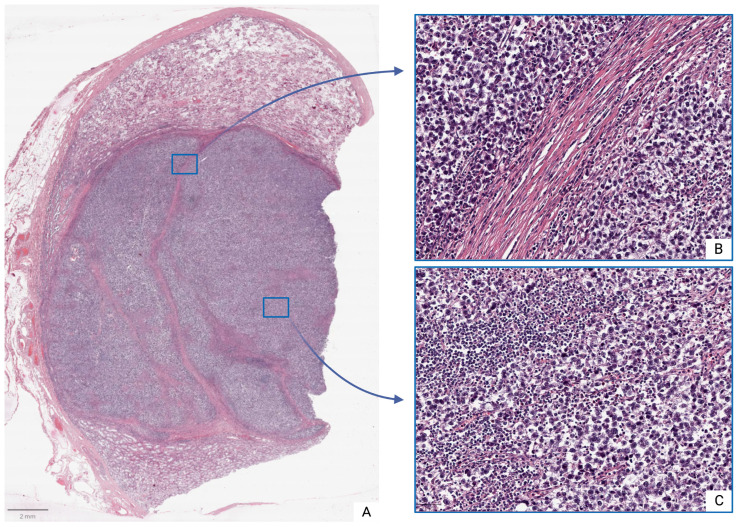
Seminoma, subgroup Ib—pT2: (**A**)—histoscan; (**B**)—stroma; (**C**)—tumor. Staining: hematoxylin and eosin, magnification ×200.

Moderate inflammatory infiltrates were observed in both tumor and peritumoral tissues, consisting of clusters of lymphocytes, plasma cells, and other immune-competent cells. Intercellular edema, microcyst formation, and foci of coagulative necrosis were also observed, particularly in the tunica albuginea, which frequently exhibited inflammatory cell infiltration (Figure 1).

In histological sections from subgroup Ia, the morphology corresponded to seminoma stage pT1: the tumor was confined to the testis and rete testis, without evidence of vascular or lymphatic invasion. In some patients (*n* = 14; 45.2%), focal invasion into the tunica albuginea was observed, though without extension into the tunica vaginalis.

In contrast, subgroup Ib seminomas were characterized by the presence of vascular or lymphatic invasion, with tumor infiltration into both the tunica albuginea and tunica vaginalis.

Samples from morphologically intact testes (Group II) demonstrated normal histoarchitecture with physiological spermatogenesis and only occasional immune cells present.

### 3.2. Immunohistochemical Assay

In seminoma tumor tissues, macrophages are represented by large cells with round, oval, or irregular shapes and abundant, finely granular or foamy cytoplasm. Their nuclei are typically eccentrically located and round or kidney-shaped, with a loose chromatin structure and one or more prominent nucleoli.

Morphologically, CD68^+^ TAMs exhibit well-defined cellular borders and a variable cell body shape, ranging from rounded to polygonal. In contrast, CD163^+^ TAMs are relatively large, with oval or irregularly shaped nuclei that are often hyperchromatic, and display abundant cytoplasm with prominent granular staining.

In normal testicular tissue, CD68^+^ and CD163^+^ macrophages are predominantly localized within the interstitial regions and exhibited variable frequencies. Quantitative analysis revealed that the number of CD68^+^ macrophages in normal testicular tissue was significantly higher than that of CD163^+^ macrophages (*p* < 0.001). The median number of CD68^+^ macrophages was approximately 120 times greater than that of CD163^+^ macrophages (Table 1).

Seminoma samples also demonstrated a significant increase in the number of macrophages of both phenotypes compared to normal tissue (*p* < 0.001 for all comparisons). Specifically, the median number of CD68^+^ TAMs in pT1- and pT2-stage tumors was approximately 5.4 and 5.6 times greater than that in the control group, respectively. CD163^+^ TAMs exhibited an even more pronounced increase: their median values in pT1- and pT2-stage seminoma exceeded those in the control group by about 23 and 79 times, respectively (Table 1).

**Figure 2:**
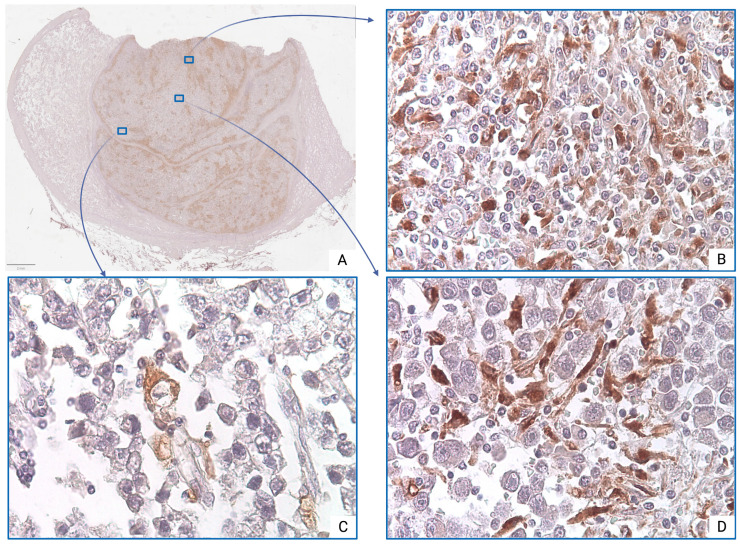
Seminoma, subgroup Ib—pT2: (**A**)—histoscan; (**B**)—diffuse infiltration; (**C**)—single cells; (**D**)—focal clusters. The CD68^+^ macrophages display intense cytoplasmic staining. Immunohistochemical reactions with anti-CD68 antibodies, magnification ×600.

Immunohistochemical analysis using anti-CD68 antibodies revealed pronounced heterogeneity in macrophage infiltration—both between cases and within individual tumor samples. The majority of CD68^+^ TAMs were located in peripheral regions of the tumor mass, where they formed dense peristromal clusters. Three major distribution patterns were identified: diffuse (Figure 2B), focal clusters (Figure 2D), and single cells (Figure 2C).

However, comparative quantitative analysis of CD68^+^ TAMs revealed no statistically significant difference in values between pT2 and pT1 stages (*p* = 0.423) (Table 1).

**Figure 3:**
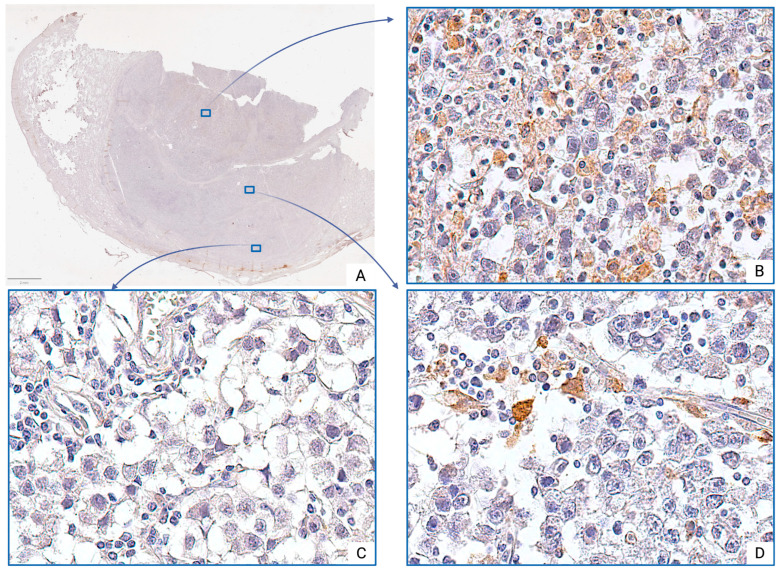
Seminoma, subgroup Ib—pT2: (**A**)—histoscan; (**B**)—diffuse infiltration; (**C**)—absence of cells; (**D**)—single cells. The CD163^+^ macrophages display intense cytoplasmic staining. Immunohistochemical reactions with anti-CD163 antibodies, magnification ×600.

CD163^+^ TAMs also demonstrated significant regional variability. Increased density of CD163^+^ TAMs was primarily observed in the central tumor regions, where they formed diffuse infiltration (Figure 3B), often associated with small-caliber blood vessels or areas of fibrotic remodeling. Single M2 macrophages were also detected in peristromal regions and around medium-sized vessels (Figure 3D). Notably, in some peripheral tumor zones, CD163^+^ TAMs were entirely absent in several visual fields (Figure 3C).

However, comparative quantitative analysis revealed that the number of CD163^+^ TAMs at the pT2 stage was significantly higher than at the pT1 stage (*p* < 0.001). The median number of CD163^+^ TAMs at the pT2 stage was approximately 3.4 times greater than that at the pT1 (Table 1).

**Figure 4:**
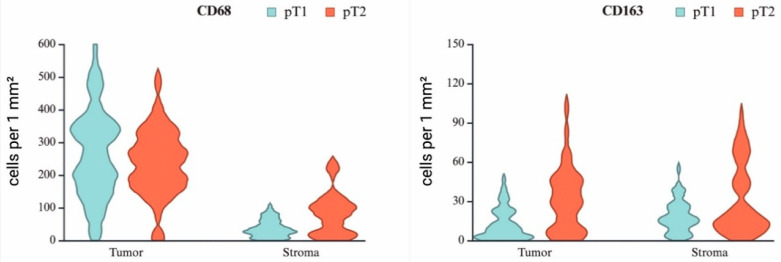
Violin plots showing the relative number of macrophages expressing CD68 and CD163 in the tumor component and stroma of seminoma at stages pT1 and pT2.

Comparative immunohistochemical analysis of CD68^+^ and CD163^+^ TAMs between pT1- and pT2-stage seminomas revealed pronounced interindividual variability, as reflected by the interquartile range (Q1–Q3) and visualized in the violin plot (Figure 4) (Table 1).

**Figure 5:**
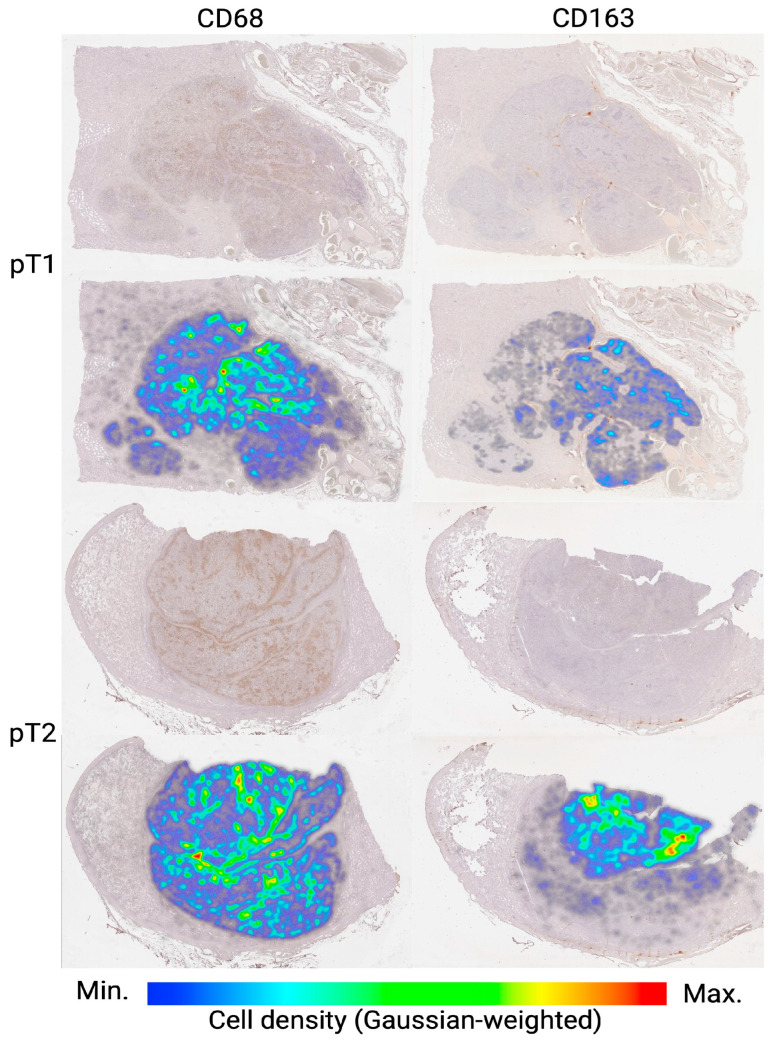
Density maps of CD68^+^ and CD163^+^ TAMs in seminoma tissue at stages pT1 and pT2. Representative whole-slide images are shown in the top rows, with corresponding density heatmaps overlaid in the bottom rows. Color bar indicates macrophage density (cells/mm^2^), normalized via Gaussian weighting (blue = minimum; red = maximum). Notably, pT2 seminomas exhibit more pronounced central clusters of CD163^+^ TAMs compared to pT1, indicating stage-dependent spatial reorganization of the TAMs microenvironment.

In addition to the quantitative increase in CD163^+^ TAMs at the pT2 stage, a notable feature was their tendency to concentrate more densely in the central region of the tumor, as clearly illustrated by the density map (Figure 5).

## 4. Discussion

**Figure 6:**
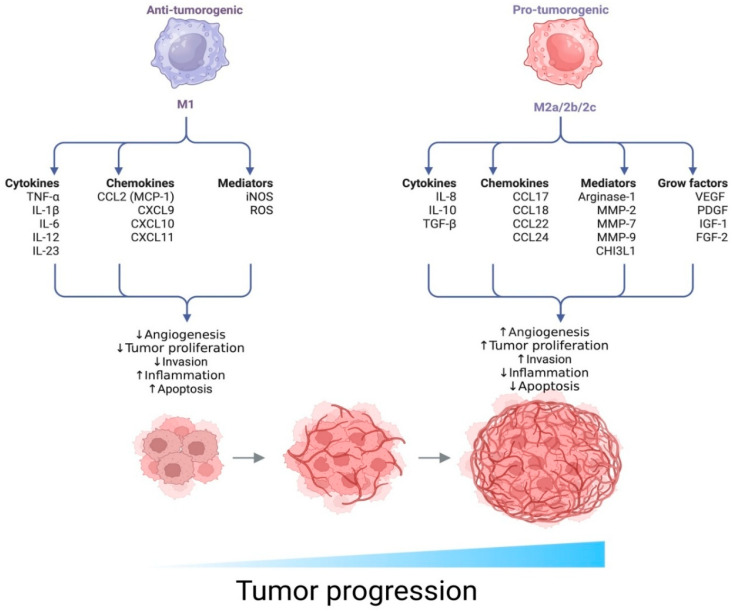
Functional polarization of macrophages and its impact on tumor progression. The diagram illustrates two opposing macrophage phenotypes—M1 and M2 (subtypes M2a/2b/2c)—which exhibit distinct biological functions within the tumor microenvironment (TME).

This study is the first to quantitatively and phenotypically characterize CD68^+^ and CD163^+^ tumor-associated macrophages (TAMs) in N0-stage seminomas at pT1 and pT2 stages. Macrophages are highly plastic effector cells of the innate immune system, derived from monocytes, and are capable of differentiating into various functional phenotypes depending on the surrounding microenvironment. Classical activation (M1 phenotype) is induced by interferon-γ and lipopolysaccharide (LPS), leading to the secretion of pro-inflammatory mediators (such as TNF-α, IL-1β, and IL-6) and the production of reactive oxygen species. In contrast, alternative activation (M2 phenotype) is induced by cytokines such as IL-4, IL-10, and IL-13 and is associated with tissue repair, angiogenesis, and immunosuppressive effects (Figure 6) [19].

The density and phenotype of macrophages vary substantially across tumor types, including germ cell tumors. For example, Sadigh et al. (2020) reported that the proportion of M2-polarized TAMs is higher in seminomas than in other germ cell tumors and that these cells often co-express PD-L1, potentially indicating an immunosuppressive tumor microenvironment. However, they did not identify a statistically significant association between TAM density and tumor stage, likely due to the limited cohort size [17].

Similar trends have been reported in other malignancies, including melanoma (Falleni et al., 2017), colorectal cancer (Edin et al., 2012), and ovarian cancer (Nowak et al., 2020), where advancing tumor stage is associated with a shift toward M2-dominated macrophage populations, typically linked to poor prognosis [20,21,22].

In the present study, no statistically significant quantitative differences in CD68^+^ TAMs were observed between pT1 and pT2 stages. However, there was a noted trend toward an increased proportion of M2 macrophages at the pT2 stage. These findings warrant further investigation, particularly regarding potential correlations with recurrence rates, given the immunosuppressive nature of M2 macrophages. For example, in a study on colorectal cancer by Xue et al. (2021), high infiltration of CD163^+^ TAMs was identified as an unfavorable prognostic factor and was associated with reduced overall survival [23].

TAMs—predominantly of the M2 phenotype—play a critical role in angiogenesis, extracellular matrix remodeling, and immunosuppression, thereby facilitating tumor progression and metastasis. The distribution of CD163^+^ TAMs observed in this study, with predominant localization in the central regions of the tumors, is consistent with previous findings suggesting that their activity is modulated by hypoxia and acidosis. These microenvironmental factors promote M2 polarization and stimulate the secretion of pro-inflammatory and pro-angiogenic mediators, such as VEGF, IL-10, and TGF-β. Consequently, M2 macrophages are regarded as potential biomarkers of tumor aggressiveness and therapeutic targets, particularly in combination with HIF inhibitors, metabolic agents, or immunotherapeutic strategies targeting the EGFR/VEGFR signaling pathways [24,25].

Recent research has also explored blocking the CCL2/CCR2 signaling axis, responsible for monocyte recruitment and M2 differentiation, as a promising strategy to reduce TAM infiltration in the TME and enhance anti-tumor immune responses. This has been supported by both preclinical models and clinical trials involving agents such as carlumab and PF-04136309 [26,27,28].

Given the marked heterogeneity of the immune infiltrate, a promising avenue for further research includes the use of multiplex fluorescent immunohistochemistry to delineate spatial interactions between M1 and M2 macrophages and other components of the TME, as well as key signaling pathways involved in macrophage polarization, including STAT1 and NF-κB.

## 5. Limitation

This study has several limitations. First, we deliberately focused on pT1 and pT2 seminomas, as these stages account for approximately 80–85% of all stage I seminoma cases. They represent the most typical and clinically relevant patient cohort for which clear criteria justifying adjuvant chemotherapy following orchifuniculectomy have not yet been established.

Second, at this stage of our investigation, we concentrated on evaluating the clinical relevance of a highly reproducible marker suitable for routine oncopathological practice—CD163. A more in-depth exploration of the tumor microenvironment, incorporating multiplex immunohistochemistry and, potentially, molecular genetic techniques, is planned for future stages of this research.

Third, it is currently not possible to perform a reliable assessment of progression-free survival, as cohort enrollment was completed only in January 2025, and the current follow-up period is insufficient to identify recurrence events.

## 6. Conclusions

The results of this study demonstrate that non-metastatic pT2-stage seminomas exhibit an increased proportion of M2 macrophages, accompanied by their focal accumulation in the central region of the tumor. This finding may serve as an additional criterion for patient stratification.

Further research employing multiplex immunohistochemistry and progression-free survival analysis is required to provide deeper insight into the mechanisms underlying seminoma progression. These investigations may help clarify whether the observed increase in M2 macrophages reflects immunosuppressive remodeling of the tumor microenvironment (TME), thereby supporting their consideration as potential targets for personalized therapeutic strategies.

## Figures and Tables

**Table 1:** Quantitative characterization of CD68 and CD163 macrophages in seminoma and normal testis per 1 mm^2^.

Marker	Group I (Seminoma)			Group II (Normal Testis) Me [Q1–Q3]
	Stage	Tumor Me [Q1–Q3]	Stroma Me [Q1–Q3]	
**CD68**	pT1	237.665 [166.648–345.462]	32.185 [20.415–55.340]	43.900 [35.410–54.380]
	pT2	247.575 [177.838–304.873]	87.945 [19.300–112.105]	
	*p*-value	0.423 *	0.001 *	
**CD163**	pT1	8.715 [1.843–21.125]	16.176 [9.977–25.670]	0.371 [0.265–0.647]
	pT2	29.327 [9.677–45.539]	19.615 [9.971–45.901]	
	*p*-value	0.001 *	0.082 *	0.001 **

*—*p*-value for comparison between pT1 and pT2 seminomas. **—*p*-value for comparison between CD68 and CD163 normal testis.

## Data Availability

The study did not generate publicly available archival data.
